# Risk Charts Illustrating the 10-year Risk of Stroke among Residents of Japanese Rural Communities: The JMS Cohort Study

**DOI:** 10.2188/jea.JE20080092

**Published:** 2009-03-19

**Authors:** Shizukiyo Ishikawa, Masatoshi Matsumoto, Kazunori Kayaba, Tadao Gotoh, Naoki Nago, Akizumi Tsutsumi, Eiji Kajii, the Jichi Medical School (JMS) Cohort Study Group

**Affiliations:** 1 Division of Community and Family Medicine, Center for Community Medicine, Jichi Medical University, Shimotsuke, Tochigi, Japan; 2 School of Health and Social Services, Saitama Prefectural University, Koshigaya, Saitama, Japan; 3 Wara National Health Insurance Clinic, Gujo, Gifu, Japan; 4 Community Medicine Education Center, Japan Association for Development of Community Medicine, Tokyo, Japan; 5 Occupational Health Training Center, University of Occupational and Environmental Health, Kitakyushu, Fukuoka, Japan

**Keywords:** stroke, blood pressure, smoking, diabetes mellitus, cohort study

## Abstract

**Background:**

Risk charts are used to estimate the risk of cardiovascular diseases; however, most have been developed in Western countries. In Japan, currently available risk charts are based on mortality data. Using data on cardiovascular disease incidence from the JMS Cohort Study, we developed charts that illustrated the risk of stroke.

**Methods and Results:**

The JMS Cohort Study is a community-based cohort study of cardiovascular disease. Baseline data were obtained between 1992 and 1995. In the present analysis, the participants were 12 276 subjects without a history of stroke; the follow-up period was 10.7 years. Color-coded risk charts were created by using Cox’s proportional hazards models to calculate 10-year absolute risks associated with sex, age, smoking status, diabetes status, and systolic blood pressure. The risks of stroke and cerebral infarction rose as age and systolic blood pressure increased. Although the risk of cerebral hemorrhage were generally lower than that of cerebral infarction, the patterns of association with risk factors were similar.

**Conclusion:**

These risk charts should prove useful for clinicians and other health professionals who are required to estimate an individual’s risk for stroke.

## INTRODUCTION

Cardiovascular disease (CVD) and cerebrovascular disease are the second and third most common causes of death in Japan.^1^ In most Western countries, the incidence of myocardial infarction (MI) is higher than that of stroke.^2^^,^^3^ However, in Japan the incidence of stroke is much higher than that of MI.^1^^,^^4^^,^^5^ Although stroke mortality^1^ and incidence have declined in the last few decades,^1^^–^^3^ stroke remains a significant healthcare burden for Japan.

Age, sex, blood pressure, smoking status, and diabetes status are considered the major factors in quantifying stroke risk.^4^^,^^6^^–^^11^ Several models to predict the risk of CVD were developed after the Framingham risk estimates were reported.^12^^–^^19^ However, most of these only assess the risk for coronary heart disease (CHD); only a few address the risk of stroke. Because the incidences of CHD and stroke differ between Japan and Western countries, risk assessment tools from the latter are not ideal for use in Japan.

Recently, a risk assessment chart for CVD was developed using data from the NIPPON DATA80 in Japan.^20^^,^^21^ However, NIPPON DATA80 investigated stroke mortality only, ie, non-fatal strokes were not included in the analysis. Therefore, a risk chart constructed using these data would not be entirely suitable for predicting stroke incidence. The Jichi Medical School (JMS) Cohort Study is a multi-community prospective study that monitors residents of Japanese rural communities and captures CVD events. We used data from the JMS Cohort Study to develop charts that display the risk of stroke among Japanese.

## METHODS

### Study population

The JMS Cohort Study began in 1992. Its primary objective was to clarify associations between potential risk factors and CVD in 12 rural districts in Japan.^5^^,^^22^

The baseline data of this cohort study were obtained between April 1992 and July 1995. If several sets of data were obtained for a single identical participant during that period, the first set was used as baseline. The baseline data were collected as a part of a national mass-screening program. In Japan, mass screening for CVD has been conducted since 1982, in accordance with the Health and Medical Service for the Aged Act of 1981. Local government offices in each community issued invitations to residents eligible for the mass screening, and personal invitations were also sent to all potential participants by mail. As a result, 12 490 participants were eligible (4913 males and 7577 females; age range, 19–93 years). The overall response rate among the 12 communities was 65.0%. Written informed consent to participate in the study was obtained individually from all the respondents to the mass screening.

Among the 12 490 participants, 95 (0.8%) who did not sign the agreement to participate in the study, 7 (0.06%) who had no follow-up data, and 112 (0.9%) who had a past history of stroke were excluded. Ultimately, 12 276 participants (4807 men and 7469 women) remained for analysis.

## Measurement of baseline variables

To ensure uniform data collection, we established a central committee composed of the chief medical officers from all the participating districts. This committee developed a detailed manual for data collection. Systolic blood pressure and diastolic blood pressure were measured once with a fully automated sphygmomanometer, the BP203RV-II (Nippon Colin, Komaki, Japan), placed on the right arm of a seated participant who had rested in a sitting position for 5 minutes before measurement. Information about medical history and lifestyle was gathered by means of a written questionnaire.

Blood samples were drawn from the antecubital vein of seated participants, with minimal tourniquet use. Specimens were collected in siliconized vacuum glass tubes containing a 1/10 volume of 3.8% trisodium citrate for blood glucose, and no additives for lipids. Tubes were centrifuged at 3000g for 15 minutes at room temperature. After separation, the serum samples were stored at 4 °C in refrigerated containers if analysis was to be performed within a few days. Otherwise, the samples were frozen until analysis. Plasma samples were frozen as rapidly as possible to −80 °C for storage, until laboratory examination could be performed.

Total cholesterol was measured by using an enzymatic method (Wako, Osaka, Japan; interassay coefficient of variation (CV): 1.5%). Blood glucose was measured via an enzymatic method (Kanto Chemistry, Tokyo, Japan; interassay CV: 1.9%). In this study, blood samples of 5547 (45.0%) participants were collected after an overnight fast; all other samples were casual samples. Diabetic participants were defined as those with currently treated diabetes, plasma glucose ≥126 mg/dl after an overnight fast, or casual blood glucose ≥200 mg/dl. Participants were also asked whether they were current smokers or not.

### Follow-up

A mass screening system was used to obtain baseline data and to follow the participants annually. Those examined were asked whether they had suffered a stroke after enrolling. Participants who did not come to the screening examination were contacted by mail or phone. Public health nurses also visited the participants to obtain pertinent information when necessary. In total, 100% of the participants were contacted. Those with a history of stroke were asked when the stroke had been diagnosed and where (at which hospital) they had been treated. Medical records at hospitals in the study areas were also checked to determine if these participants had been treated. If an incident was suspected, forms were filled out, and duplicates of the CT and/or MRI films of the case were obtained to confirm a diagnosis of stroke. Diagnoses were determined independently by a diagnosis committee comprising 1 radiologist, 1 neurologist, and 2 cardiologists. Stroke was defined as a focal, nonconvulsive neurological deficit of sudden onset that persisted for at least 24 hours. Stroke subtypes, ie, cerebral hemorrhage (CH), cerebral infarction (CI), and subarachnoid hemorrhage (SAH), were determined by using the criteria of the National Institute of Neurological Disorder and Stroke.^23^ Symptomatic lacuna infarction was defined as a CI.

### Statistical analysis

Statistical analyses were carried out using SAS version 8.2 (SAS Japan). Cox proportional hazards models were used to calculate the 10-year absolute risk of stroke for each risk factor. Under the Cox proportional hazards model, the survival probability S(T:X) of a person with a risk X at time T is defined as S(T:X) = {[S0(T)]exp^(BX)}exp(B(X−Xm))^, where S0(T) is survival probability corresponding to the standard hazard, B is the regression coefficient, and Xm is the population mean of risk X. The 10-year absolute risk of a person with risk X is thus 1-S(10:X).^21^ Risk charts were created based on calculations of the absolute risk associated with 5 conventional cardiovascular risk factors: age, sex, smoking status, diabetes status, and systolic blood pressure. Age was grouped into 5 categories: less than 40, 40–49, 50–59, 60–69, and 70 years or older. Systolic blood pressure was also grouped into 5 categories: less than 120, 120–139, 140–159, 160–179, and 180 mm Hg or higher. The other risk factors were treated as dichotomous variables. The risk charts were color-coded so that users could easily estimate their probability of a stroke.

## RESULTS

The mean age of participants at baseline was 55.2 years for men and 55.3 years for women. The mean duration of follow-up was 10.7 years (men: 10.6 years; women: 10.8 years). Total incidence of stroke was 190 cases for men (CH: 41 [21.6%], CI: 136 [71.6%], SAH: 13 [6.8%]) and 165 cases for women (CH: 38 [23.0%], CI: 87 [52.7%], SAH: 39 [23.6%], Unclassified: 1 (0.6%]) (Table 1).

**Table 1.** Participants from JMS Cohort Study included in the analysis of stroke risk

	Men	Women
Total Cohort Participants	4911	7579
Participants with Consent	4869	7519
Study Participants	4406	6817
Duration of follow-up (years)	10.6 ± 2.6	10.8 ± 2.2
Age (years)	55.2 ± 12.0	55.3 ± 11.2
Systolic Blood Pressure (mm Hg)	131.3 ± 20.5	128.0 ± 21.0
Smoker (%)	50.4	5.5
Diabetes (%)	4.5	2.2
Stroke	190	165
Cerebral hemorrhage	41	38
Cerebral infarction	136	87
Subarachnoid hemorrhage	13	39
Unclassified	0	1

**Figure 1.**
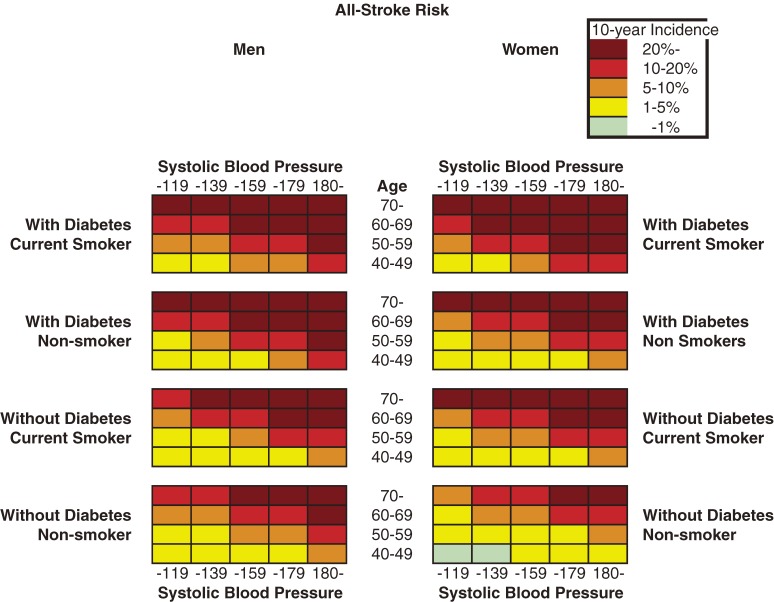
Chart showing 10-year all-stroke risk in men and women

**Figure 2.**
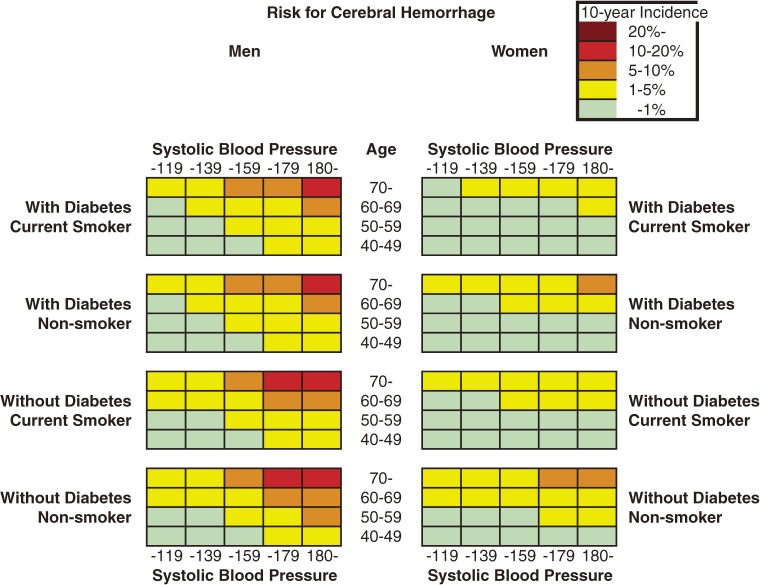
Chart showing 10-year risk for cerebral infarction in men and women

**Figure 3.**
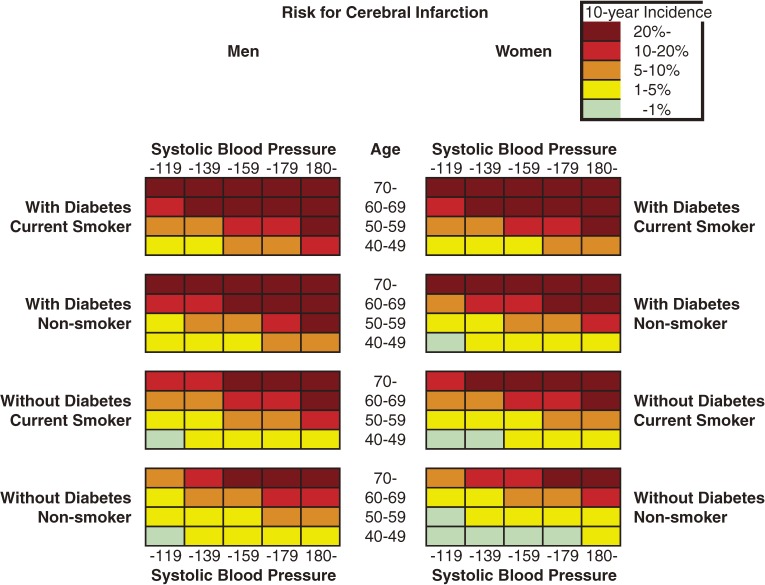
Chart showing 10-year risk for cerebral hemorrhage in men and women

Figures 1 to 2 show the color-coded 10-year absolute risk for all stroke, CH, and CI for each of the combinations of risk factors. All charts were prepared in the same manner, according to diabetes status, smoking status, and systolic blood pressure in each sex. Initially, total cholesterol was included in the analysis; however, after it was determined that total cholesterol was not associated with stroke in either sex, it was excluded from the model. Figure 1 shows all-stroke risk in both sexes, Figure 3 shows CH risk, and Figure 2 shows the risk for CI. Risk can be read by matching the individual’s age to the appropriate age group, and blood pressure to the nearest multiple of 20 mm Hg. The risks rose as systolic blood pressure and age increased. In addition, among men and women, current smokers and participants with diabetes were at higher risk for any stroke event and for CI. Although the 10-year risk of CH was lower than that of CI, the risk patterns were similar in men. The risk of SAH was higher in women than in men, and was positively associated with SBP in women, but not in men. The SAH data in the present study are not shown because the number of cases was small, especially among men, and thus the charts were not likely to be representative.

## DISCUSSION

We developed risk charts for stroke based on data from the JMS Cohort Study. The charts show 10-year absolute risk for all stroke events and stroke subtypes associated with sex, age, smoking status, diabetes status, and systolic blood pressure. Because cholesterol was not associated with stroke, it was not included as an independent variable in the analysis. We believe that these charts will be useful for clinicians and other health professionals who are required to estimate an individual’s risk for stroke.

The charts were developed using data from a Japanese community-based cardiovascular cohort study. In the past, a variety of risk charts were developed to estimate the probability of CVD. However, most used data from studies conducted in Western countries.^6^^,^^11^^–^^13^^,^^17^^–^^19^^,^^24^^–^^27^ Risk profile charts that estimate the probability of stroke were developed using data from the Framingham Heart Study.^6^^,^^27^ Although these risk estimates have been widely adopted in the formulation of clinical guidelines in the United States and elsewhere,^28^^–^^30^ a number of problems in applying these estimates to other populations have been described, the most important of which is that incidences of CVD substantially differ by population.^7^^,^^31^^,^^32^ More recently, tools have been developed that estimate cardiovascular risk in a number of specific populations.^11^^,^^13^^,^^17^^–^^19^^,^^25^^,^^26^

The Framingham estimates of stroke risk cannot be applied to Japanese because the incidence of stroke differs between the United States and Japan.^33^^–^^35^ In Asia, tools for CHD risk prediction that are derived from Asian cohorts have been utilized^11^; however, there have been no instruments to predict the risk for stroke in an Asian population. In Japan, cardiovascular risk charts were developed using data from the NIPPON DATA80, which is a representative cohort study. However, the charts were based on mortality data.^20^^,^^21^ The risk charts developed in the present study should be more accurate in predicting the risk for a stroke event, as opposed to stroke mortality, in Japanese.

We created sex-specific charts that showed risks associated with age, diabetes status, smoking status, and systolic blood pressure. A strong dose-response relationship between systolic blood pressure and stroke has been observed in Japan and other countries.^8^^–^^10^^,^^36^ It is important to note that in the present study total cholesterol was excluded in the estimates of stroke risk because there was no association between total cholesterol and stroke. However, charts for MI risk that are based on data from the JMS Cohort Study have been produced, and these do include total cholesterol as a risk factor for MI.

We developed 10-year charts illustrating CH and CI risk, as well as all-stroke risk. The pattern for all-stroke risk resembled that of CI risk because more than half of the recorded stroke events were CI. The chart patterns for all-stroke, CH, and CI risk were similar, among both men and women.

There were some limitations in this study. The study participants were not randomly selected. The areas in which the study was conducted were primarily rural and the data may therefore not be generalizable to urban populations. In addition, a high-risk population may benefit from more intensive monitoring and intervention than would a population at lower risk. This effect may have led to an underestimation of risks in the present study, because health promotion activities were held in the participating areas.

The strengths of the present study include its very high rates of response and follow-up. Furthermore, the present study was conducted in a standardized fashion in 12 geographically dispersed areas of Japan, and it comprised more than 12 000 men and women. These advantages substantially increased the reliability of the study results.

In conclusion, we used data from Japanese rural populations to develop risk charts that estimate the 10-year risk for stroke in individuals. However, these charts should be used with caution in non-rural populations.

## References

[1] Vital Statistics of Japan, 2000, Vol. 3. Tokyo. Health and Welfare Statistical Association 2000.

[2] Sytkowski PA , D'Agostino RB , Belanger A , Kannel WB. Sex and time trends in cardiovascular disease incidence and mortality: the Framingham Heart Study, 1950–1989. Am J Epidemiol. 1996;143:338–50. 8633618 10.1093/oxfordjournals.aje.a008748

[3] Truelsen T , Mahonen M , Tolonen H , Asplund K , Bonita R , Vanuzzo D. Trends in stroke and coronary heart disease in the WHO MONICA Project. Stroke. 2003;34:1346–52. 10.1161/01.STR.0000069724.36173.4D 12738889

[4] Kubo M , Kiyohara Y , Kato I , Tanizaki Y , Arima H , Tanaka K , Trends in the incidence, mortality, and survival rate of cardiovascular disease in a Japanese community: the Hisayama study. Stroke. 2003;34:2349–54. 10.1161/01.STR.0000090348.52943.A2 12958323

[5] Ishikawa S , Kayaba K , Gotoh T , Nago N , Nakamura Y , Tsutsumi A , Incidence of Total Stroke, Stroke Subtypes, and Myocardial infarction in Japanese Population: The JMS Cohort Study. J Epidemiol. 2008;18:144–50. 10.2188/jea.JE2007438 18603825 PMC4771583

[6] Wolf PA , D'Agostino RB , Belanger AJ , Kannel WB. Probability of stroke: a risk profile from the Framingham Study. Stroke. 1991;22:312–8. 2003301 10.1161/01.str.22.3.312

[7] D'Agostino RB Sr , Grundy S , Sullivan LM , Wilson P. Validation of the Framingham coronary heart disease prediction scores: results of a multiple ethnic groups investigation. JAMA. 2001;286:180–7. 10.1001/jama.286.2.180 11448281

[8] Psaty BM , Furberg CD , Kuller LH , Cushman M , Savage PJ , Levine D , Association between blood pressure level and the risk of myocardial infarction, stroke, and total mortality: the cardiovascular health study. Arch Intern Med. 2001;161:1183–92. 10.1001/archinte.161.9.1183 11343441

[9] Prospective Studies Collabration. Age-specific relevance of usual blood pressure to vascular mortality: a meta-analysis of individual data for one million adults in 61 prospective studies. Lancet. 2002;360:1903–13. 10.1016/S0140-6736(02)11911-8 12493255

[10] Asia Pacific Cohort Studies Collaboration. Blood pressure and cardiovascular disease in the Asia Pacific region. J Hypertens. 2003;21:707–16. 10.1097/00004872-200304000-00013 12658016

[11] Asia Pacific Cohort Studies Collaboration. Systolic blood pressure, diabetes and the risk of cardiovascular diseases in the Asia-Pacific region. J Hypertens. 2007;25:1205–13. 10.1097/HJH.0b013e3280dce59e 17563533

[12] Anderson KM , Odell PM , Wilson PW , Kannel WB. Cardiovascular disease risk profiles. Am Heart J. 1991;121:293–8. 10.1016/0002-8703(91)90861-B 1985385

[13] Conroy RM , Pyorala K , Fitzgerald AP , Sans S , Menotti A , De Backer G , Estimation of ten-year risk of fatal cardiovascular disease in Europe: the SCORE project. Eur Heart J. 2003;24:987–1003. 10.1016/S0195-668X(03)00114-3 12788299

[14] De Backer G , Ambrosioni E , Borch-Johnsen K , Brotons C , Cifkova R , Dallongeville J , European guidelines on cardiovascular disease prevention in clinical practice: Third Joint Task Force of European and other Societies on Cardiovascular Disease Prevention in Clinical Practice (constituted by representatives of eight societies and by invited experts). Eur Heart J. 2003;24:1601–10. 10.1016/S0195-668X(03)00347-6 12964575

[15] British Cardiac Society, British Hypertension Society, Diabetes UK, HEART UK, Primary Care Cardiovascular Society, The Stroke Association. JBS 2: Joint British Societies' guidelines on prevention of cardiovascular disease in clinical practice. Heart. 2005;91:v1–52. 10.1136/hrt.2005.079988 16365341 PMC1876394

[16] Menotti A , Lanti M , Agabiti-Rosei E , Carratelli L , Cavera G , Dormi A , Riskard 2005. New tools for prediction of cardiovascular disease risk derived from Italian population studies. Nutr Metab Cardiovasc Dis. 2005;15:426–40. 10.1016/j.numecd.2005.07.007 16314229

[17] Ridker PM , Buring JE , Rifai N , Cook NR. Development and validation of improved algorithms for the assessment of global cardiovascular risk in women: the Reynolds Risk Score. JAMA. 2007;297:611–9. 10.1001/jama.297.6.611 17299196

[18] Hippisley-Cox J , Coupland C , Vinogradova Y , Robson J , May M , Brindle P. Derivation and validation of QRISK, a new cardiovascular disease risk score for the United Kingdom: prospective open cohort study. BMJ. 2007;335:136–41. 10.1136/bmj.39261.471806.55 17615182 PMC1925200

[19] Woodward M , Brindle P , Tunstall-Pedoe H SIGN group on risk estimation. Adding social deprivation and family history to cardiovascular risk assessment: the ASSIGN score from the Scottish Heart Health Extended Cohort (SHHEC). Heart. 2007;93:172–6. 10.1136/hrt.2006.108167 17090561 PMC1861393

[20] Kasagi F , Kodama K , Hayakawa T , Okayama A , Ueshima H Risk appraisal chart based on NIPPON DATA80: Stroke and coronary heart disease. Jpn J Cardiovasc Dis Prev. 2005;40:20–6 (in Japanese).

[21] NIPPON DATA80 Research Group. Risk assessment chart for death from cardiovascular disease based on a 19-year follow-up study of a Japanese representative population. Circ J. 2006;70:1249–55. 10.1253/circj.70.1249 16998254

[22] Ishikawa S , Gotoh T , Nago N , Kayaba K. The Jichi Medical School (JMS) Cohort Study: design, baseline data and standardized mortality ratios. J Epidemiol. 2002;12:408–17. 12462275 10.2188/jea.12.408 PMC10681813

[23] Adams HP Jr , Bendixen BH , Kappelle LJ , Biller J , Love BB , Gordon DL , Classification of subtype of acute ischemic stroke. Definitions for use in a multicenter clinical trial. TOAST. Trial of Org 10172 in Acute Stroke Treatment. Stroke. 1993;24:35–41. 7678184 10.1161/01.str.24.1.35

[24] Anderson KM , Wilson PW , Odell PM , Kannel WB. An updated coronary risk profile. A statement for health professionals. Circulation. 1991;83:356–62. 1984895 10.1161/01.cir.83.1.356

[25] Menotti A , Lanti M , Puddu PE , Kromhout D. Coronary heart disease incidence in northern and southern European populations: a reanalysis of the seven countries study for a European coronary risk chart. Heart. 2000;84:238–44. 10.1136/heart.84.3.238 10956281 PMC1760967

[26] Assmann G , Cullen P , Schulte H. Simple Scoring Scheme for Calculating the Risk of Acute Coronary Events Based on the 10-Year Follow-Up of the Prospective Cardiovascular Munster (PROCAM) Study. Circulation. 2002;105:310–5. 10.1161/hc0302.102575 11804985

[27] Kannel WB , D'Agostino RB , Sullivan L , Wilson PW. Concept and usefulness of cardiovascular risk profiles. Am Heart J. 2004;148:16–26. 10.1016/j.ahj.2003.10.022 15215787

[28] Pyorala K , De Backer G , Graham I , Poole-Wilson P , Wood D. Prevention of coronary heart disease in clinical practice. Recommendations of the Task Force of the European Society of Cardiology, European Atherosclerosis Society and European Society of Hypertension. Eur Heart J. 1994;15:1300–31. 7821306 10.1093/oxfordjournals.eurheartj.a060388

[29] WHO Guidelines Subcommittee. 1999 World Health Organization-International Society of Hypertension Guidelines for the Management of Hypertension. Guidelines Subcommittee. J Hypertens. 1999;17:151–83. 10067786

[30] Expert Panel on Detection, Evaluation, and Treatment of High Blood Cholesterol in Adults. Executive Summary of the Third Report of the National Cholesterol Education Program (NCEP) Expert Panel on Detection, Evaluation, and Treatment of High Blood Cholesterol in Adults (Adult Treatment Panel III). JAMA. 2001;285:2486–97. 10.1001/jama.285.19.2486 11368702

[31] Liu J , Hong Y , D'Agostino RB Sr , Wu Z , Wang W , Sun J , Predictive value for the Chinese population of the Framingham CHD risk assessment tool compared with the Chinese Multi-Provincial Cohort Study. JAMA. 2004;291:2591–9. 10.1001/jama.291.21.2591 15173150

[32] Brindle P , Beswick A , Fahey T , Ebrahim S. Accuracy and impact of risk assessment in the primary prevention of cardiovascular disease: a systematic review. Heart. 2006;92:1752–9. 10.1136/hrt.2006.087932 16621883 PMC1861278

[33] Shimamoto T , Iso H , Iida M , Komachi Y. Epidemiology of cerebrovascular disease: stroke epidemic in Japan. J Epidemiol. 1996;6:S43–7. 8800273 10.2188/jea.6.3sup_43

[34] van den Hoogen PC , Feskens EJ , Nagelkerke NJ , Menotti A , Nissinen A , Kromhout D , The Relation between Blood Pressure and Mortality Due to Coronary Heart Disease among Men in Different Parts of the World. N Engl J Med. 2000;342:1–8. 10.1056/NEJM200001063420101 10620642

[35] Stamler J , Elliott P , Dennis B , Dyer AR , Kesteloot H , Liu K , INTERMAP: background, aims, design, methods, and descriptive statistics (nondietary). J Hum Hypertens. 2003;17:591–608. 10.1038/sj.jhh.1001603 13679950 PMC6660162

[36] Lawes CM , Bennett DA , Feigin VL , Rodgers A. Blood pressure and stroke: an overview of published reviews. Stroke. 2004;35:1024–33. 10.1161/01.STR.0000116869.64771.5A 15053002

